# Social Support in a Novel Situation Aimed for Stunning and Euthanasia of Pigs May Be Increased by Familiar Pigs—A Behavioural Study with Weaners

**DOI:** 10.3390/ani13030481

**Published:** 2023-01-30

**Authors:** Astrid Söderquist, Anna Wallenbeck, Cecilia Lindahl

**Affiliations:** 1 Department of Agriculture and Food, RISE Research Institutes of Sweden AB, 750 07 Uppsala, Sweden; 2 Department of Animal Environment and Health, Swedish University of Agricultural Sciences, 532 23 Skara, Sweden; 3 Department of Physics, Chemistry and Biology, Faculty of Medicine and Health Sciences, Linköping University, 581 83 Linköping, Sweden

**Keywords:** animal welfare, swine, companionship, foam, slaughter, social behaviour

## Abstract

**Simple Summary:**

None of the approved methods for stunning pigs prior to slaughter is ideal from an animal welfare viewpoint. A method involving use of high-expansion foam to encapsulate nitrogen gas has recently been proposed as an alternative humane stunning method. The method is effective, but the foam itself induces some distress to individually exposed pigs. This study evaluated the effects of companionship from a familiar or unfamiliar conspecific during air-filled foam exposure on pigs’ behavioural response. Companionship was found to be related to lower activity levels and fewer escape attempts. When comparing companionship with familiar and unfamiliar conspecifics, it was found that pig pairs with familiar individuals spent more time in physical contact during foam exposure, possibly seeking social support. The results highlight the importance of contact with conspecifics when studying animal welfare and demonstrates the potential benefits of maintaining stable familiar pig groups up to the point of stunning at slaughter.

**Abstract:**

The common method of stunning pigs using high concentration carbon dioxide prior to slaughter poses an animal welfare issue, as the gas is aversive. Proof of concept for using nitrogen gas encapsulated in high-expansion foam as an alternative non-aversive method for stunning pigs has recently been presented. However, the individually tested pigs showed distress-related responses to foam exposure, regardless of whether it was nitrogen- or air-filled. This study examined the effect of companionship from a familiar or unfamiliar pig on behaviours in 72 nine-weeks old pigs during exposure to air-filled foam. Escape attempts were observed by 75% of solitary pigs, 42% of pigs with unfamiliar conspecifics, and 33% of pigs with familiar conspecifics. Familiar pig pairs clearly preferred social contact during foam exposure, whereas this was not as clear in unfamiliar pig pairs, and their motivation for social contact could have multiple explanations. The results from this study highlight the importance of contact with conspecifics when studying animal welfare and suggest that familiarity between pigs is important for social support, thus emphasizing the importance of maintaining social groups to reduce distress in pigs at slaughter.

## 1. Introduction

There is an urgent need for less aversive methods for stunning pigs prior to slaughter [1,2,3]. The widely used carbon dioxide stunning method has the advantage of keeping pigs in groups with minimal human handling, but the adverse effect of the gas itself is a major animal welfare issue at global scale [4,5].

Recent work provided proof of concept for a novel method using high-expansion foam filled with nitrogen gas [6]. In contrast to carbon dioxide, pigs have no chemoreceptors sensitive to nitrogen gas [7], and it is, therefore, not aversive to inhale; thus, stunning with nitrogen has been suggested as a more humane alternative to carbon dioxide stunning. However, weaner pigs exposed to foam in the previous study displayed some aversive behaviours, regardless of whether the foam was filled with air or nitrogen [6]. After showing initial interest by approaching and exploring the foam with their snout, the pigs displayed aversive behaviours, such as escape attempts as the foam levels rose above their head [6]. The pigs were alone in the box during the treatment, and as isolation itself is stressful to pigs, one of the questions remaining after that study was the potential effects of social companions on the pigs’ responses to foam exposure. The nitrogen foam method may allow slaughter pigs to be stunned in groups when applied in a slaughter situation; therefore, the effect of social support is relevant when assessing the method. Furthermore, the nitrogen foam method has been suggested as an alternative method for on-farm euthanasia of piglets, as well as older pigs, enabling euthanasia of more than one individual at a time [8].

Social animals derive benefits from the companionship of conspecifics at times of stress [9]. This effect of stress attenuation through social contact is referred to as social support or social buffering, and it is suggested to be mediated by mechanisms such as release of oxytocin and endogenous opioids [10]. Its effects are known with regard to improving animal welfare-related indicators of behaviour, physiology, and neural expression [11,12,13]. Social support can also improve the immune function of animals [14,15]. Thus, providing social animals with companionship can improve animal welfare in challenging situations.

Previous studies have found that not all companions are equally effective social supporters [16,17,18,19,20,21]. For young piglets, familiarity has been shown to be beneficial, but not necessary, for social support [12,15]. Given the natural social behaviour of mature pigs, where strangers are met with exclusion or aggression [22,23], it is possible that familiarity gradually becomes more important for the benefit of social support as pigs grow older. Beyond a certain age, familiarity between pigs may even become vital for deriving social support from a companion in a novel situation.

The aims of this study were to assess the behavioural responses of pigs to a novel situation aimed for stunning and euthanasia of weaner pigs, i.e., exposure to high-expansion air-filled foam, and to evaluate the effects of social support from another familiar or unfamiliar pig. The choice of foam exposure as the novel experience is related to a potential new stunning method for slaughter pigs, where nitrogen gas in foam is used to push the air from a confined space, thus creating an anoxic atmosphere.

## 2. Materials and Methods

### 2.1. Equipment Setup

**Figure 1:**
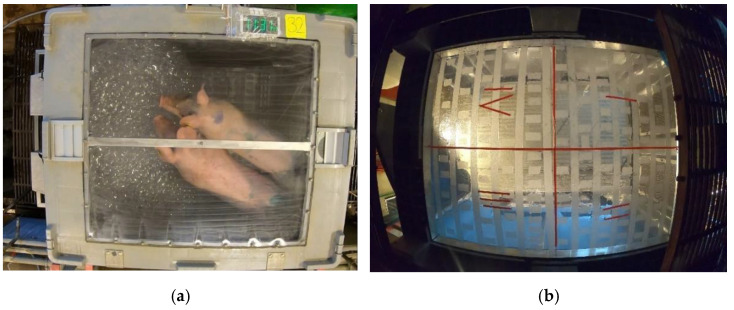
Camera angles used for recording pig behaviour through (**a**) the transparent lid and (**b**) the floor of the box, divided into four equal squares (I-IV) with coloured tape.

A specially designed foam-generating box with the measurements 110 × 92 × 67 cm, produced by the Dutch company Anoxia B.V., was used in the study. The same box was used in a previous study [6]. Two foam generators were mounted on one wall inside the box. Two bottles of compressed air (200 bar; AirLiquide gas AB, Uppsala, Sweden) and a liquid tank with a premixed foam solution (water and 3% foam agent (HTF-1000, Dr. R. Sthamer GmbH & Co., Hamburg, Germany)) was connected to the box by tubes. The high-expansion foam was produced by nozzles spraying foam solution on a metal mesh and then pushing air (7 bar pressure) through the mesh to create bubbles. The box was also equipped with a gas pulse system to destroy the foam, once the box was completely filled, to increase visibility. The box floor and lid were transparent to enable video recordings of the pigs from above and below. Anti-slip tape was used on the floor to prevent pigs from slipping when the floor became wet. Furthermore, the floor was divided into four squares of equal size, marked with tape strips (Figure 1b).

Two cameras (GoPro 7 black, GoPro, San Mateo, CA, USA) were used to video-record the pigs in the foam box from two different angles (Figure 1a,b). The foam box was placed over a culvert, enabling filming from under the box by placing one camera in the culvert. The second camera filmed through the transparent lid of the box and was live-streamed to a smartphone to allow monitoring of the pigs in real time without interfering with the experiment. A microphone was placed inside the box and connected to the camera in the culvert. For identification purposes, each test was randomly assigned a test number, which was written on two adhesive labels attached to the lid and underside of the box at points visible on the video recordings. This helped in pairing the videos from the two cameras for each test. A clicker marked the moment in time when the door of the box was closed behind a pig or pair of pigs, and then the acclimatisation period started, which facilitated synchronization of the videos from the two cameras.

### 2.2. Pigs and Housing

The experiments were conducted at the Swedish Livestock Research Centre, Swedish University of Agricultural Sciences, Uppsala, Sweden. A total of 72 pigs (crossbreeds, Yorkshire × Hampshire) of approximately 9 weeks of age (mean age 66.3 ± 3.4 days), from 11 different litters and 4 production batches, were used in the study.

The pigs were born and raised at the research facility. Cross-fostering was carried out when essential for piglet survival, but no cross-fostered pigs were used in the experiments. The pigs were weaned at five weeks of age, and pig groups remained unchanged after weaning. The pigs remained in the nursing pens without the mother sow for an additional five weeks after weaning. Feed and water were available ad libitum, and the pens were cleaned and enriched with chopped straw daily. The pigs were weighed at 9 weeks of age, about a week (1–8 days) prior to taking part in the experiment (Table 1).

The 72 pigs were allocated to 6 treatments involving foam exposure and social environment (Control Alone, Control Familiar pair, Control Unfamiliar pair, Foam Alone, Foam Familiar pair, and Foam Unfamiliar pair), with 12 pigs per treatment. Assignment to treatment was done by randomly grouping pigs within litter and sex into groups of three and then randomly selecting one pig for each of the three treatments. The next group of three pigs was randomly assigned to each of the three remaining treatments, and this was repeated until all pigs were assigned to a treatment. Pigs assigned to pair treatments (Control Familiar pair, Control Unfamiliar pair, Foam Familiar pair, Foam Unfamiliar pair) were randomly paired together so that pigs within the same litter were paired in the familiar pair treatments (Control Familiar pair and Foam Familiar pair) and pigs from two different litters were paired in the unfamiliar pair treatments (Control Unfamiliar pair and Foam Unfamiliar pair). Pig pairs consisted of one male and one female pig.

### 2.3. Experimental Procedure

The experiments were conducted during six days in November and December 2019, and the study period was divided into four sections, one for each production batch, with pigs from 2–3 litters used per production batch.

Before the experiments started, pigs from the same litter were moved to a pen in an otherwise empty part of the facility, where they were allowed to acclimatise for at least 15 min. The pigs were provided with straw, feed, and water ad libitum in the temporary pens.

For identification purposes, the pigs were marked with different colours of marking spray on their back just prior to the experiment. The test pig or pig pair were moved to the foam box and allowed to acclimatise for 2 min inside the foam box before the treatment started. Between each test pig or pig pair, the box was rinsed clean with water. Pigs fighting, i.e., engaged in prolonged agonistic behaviour, was set as a humane endpoint.

The treatments were based on a combination of two factors: foam exposure and social environment.

The two exposure conditions were:

- Control—The pig/pig pair was kept in the box for another 5 min after the acclimatisation period.
- Foam—Foam production was initiated after the acclimatisation period and turned off when the box was filled with foam. The box with foam was left for 10 s, whereafter the foam was dissolved with an air pulse. Five minutes after the start of foam production, the treatment was completed, and the pig/pig pair was let out of the box.
- The three social environment conditions were:
- Alone—The pig was in the box alone.
- Familiar pair—The pig was in the box with a pig of the opposite sex from the same litter.
- Unfamiliar pair—The pig was in the box with an age-matched pig of the opposite sex from another litter.

### 2.4. Behavioural Observations

The behavioural observations were carried out from the video recordings. The behaviour of the pigs was registered according to the ethogram in Table 2 by one observer watching the videos from both above and below the foam box. The identity of the pig (i.e., sex and familiarity between pig pairs), which was unknown to the observer during the analysis of behaviour, was registered by data based on test number and pig spray-marking colour. The video recordings from below were used to observe the position of the pigs’ hooves and to score vocalisations, while the video recordings from above were used to observe the direction of escape attempts and to observe most social behaviours.

In the foam treatments (Foam Alone, Foam Familiar pair, and Foam Unfamiliar pair), the behavioural observation period was 120 s, beginning 30 s before the start of foam production. For controls (Control Alone, Control Familiar pair, and Control Unfamiliar pair), behaviours were observed for a corresponding period, i.e., the final 30 s of the acclimatisation period and a subsequent period of 90 s. All behaviours except “Agonistic” were recorded continuously per 10 s interval, resulting in 12 intervals per test. Agonistic behaviours were rare and only measured as an estimate of severity per test.

### 2.5. Statistical Analyses

The data obtained (Supplementary Materials, Table S1) were partly edited before statistical analyses. Escape attempts (escape door, escape roof, and escape wall) rarely occurred and were, therefore, combined into a binary variable representing whether escape attempts occurred or not within each interval. Vocalisation scores were pooled into a binary variable expressing whether low-frequency vocalisations (i.e., grunting) or high-frequency vocalisations (i.e., grunt–squeals, squeals, or screams) occurred or not within each interval. Agonistic behaviours were not statistically analysed due to low occurrence. Due to failure of the equipment, one test from the treatment Control Alone was excluded from the analysis of vocalisations.

The behavioural data were divided into 10 s time intervals, where intervals 1–3 were pre-treatment and intervals 4–12 were during foam exposure for the three foam treatments. The breaking of foam occurred in different intervals, depending on the actual time it took to fill the box.

Statistical analyses were performed using Statistical Analysis Software (SAS) version 9.4 (SAS Institute Inc., 2012). Descriptive statistics were calculated using Proc Means and Proc Freq, the later including the Chi-square test. Differences between treatments were compared within each 10 s interval and between intervals. For continuous variables, residuals were assessed for normal distribution using Proc Univariate, considering the Shapiro–Wilk test for normality and a normal probability plot. The normally distributed variables were activity (i.e., the number of floor squares crossed in the box), closeness (i.e., the time pigs spent sharing a floor square inside the box), and contact (i.e., the time pigs spent in physical contact), which were analysed with general linear models 1 and 2 in Proc Mixed. Model 1 was used for the comparison of activity across all six treatments, and Model 2 was used for the comparison of pair treatments (Control Familiar pair, Control Unfamiliar pair, Foam Familiar pair, and Foam Unfamiliar pair):

Model 1: y = control foam + alone familiar unfamiliar + interval 10 s + sex + litter + control foam × alone familiar unfamiliar + control foam × interval 10 s + alone familiar unfamiliar × interval 10 s + control foam × alone familiar unfamiliar * interval 10 s + weight at 9 weeks + e

Model 2: y = control foam + alone familiar unfamiliar + interval 10 s + sex + litter + control foam ×alone familiar unfamiliar + control foam × interval 10 s + alone familiar unfamiliar × interval 10 s + control foam × alone familiar unfamiliar × interval 10 s + weight at 9 weeks + weight difference in pair + e

The binary variables (behaviour observed or not during the 10 s interval) escape attempts, low-frequency vocalisations, and high-frequency vocalisations were analysed with the generalised linear Model 3 in Proc Glimmix (using binominal distribution and logit link):

Model 3: y = control foam + alone familiar unfamiliar + interval 10 s + sex + control foam × alone familiar unfamiliar + control foam × interval 10 s + alone familiar unfamiliar × interval 10 s + weight at 9 weeks + e

In all three models, control foam (2 classes), alone familiar unfamiliar (3 classes), and interval 10 s (intervals 1–12) were included as fixed effects; litter was included as a random effect; and weight at 9 weeks and weight difference in the pair were included as continuous covariates. The three-way interaction control foam * alone familiar unfamiliar * interval 10 s was excluded from model 3, as it was not significant for any of the analysed dependent variables.

## 3. Results

### 3.1. Filling Time of Foam in Box

The mean duration to fill the box with foam was 53 ±10 s for Foam Alone, 55 ± 11 s for Foam Familiar pair, and 51 ±6 s for Foam Unfamiliar pair. Pairwise comparisons with *t*-test showed no significant difference in the foam filling time between the treatments.

### 3.2. Pig Behaviour

**Figure 2:**
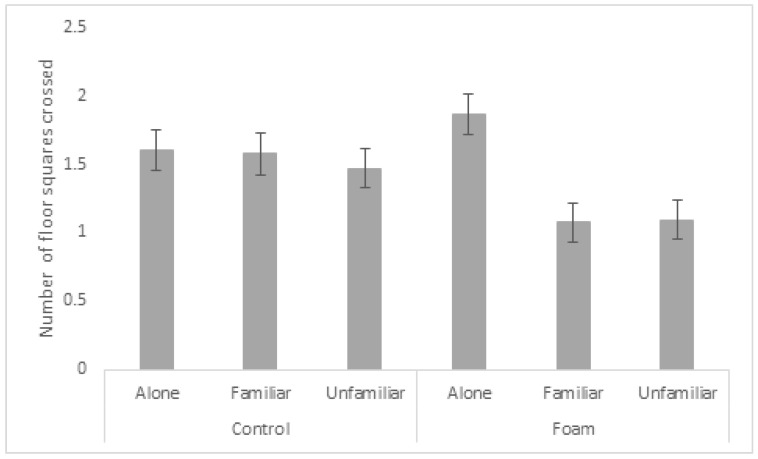
Activity within the foam box described by mean number of floor squares crossed by pigs in the six foam and control treatments. Values given are least squares mean (LSM) ± standard error (SE). Lack of overlap of SE error bars indicates significant pairwise differences (*p* < 0.05) between treatments. Overall *p*-value for the interaction: *p* < 0.001.

**Figure 3:**
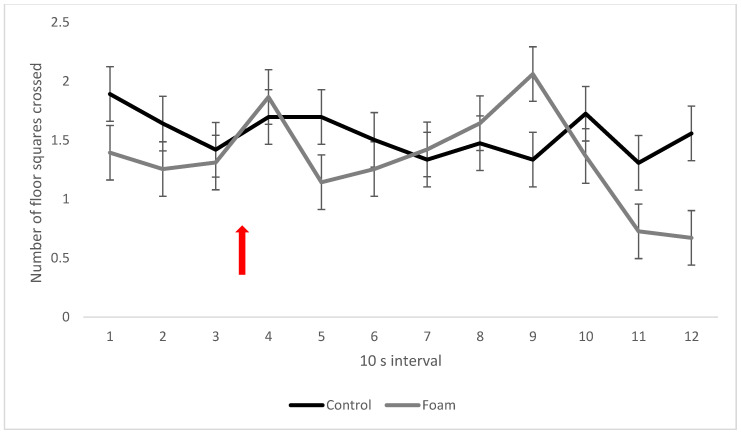
Activity within the foam box described by mean number of floor squares crossed by pigs per 10 s interval for the control and foam treatments. Red arrow indicates time of foam start. Values given are LSM ± SE. Lack of overlap of SE error bars indicates significant pairwise differences (*p* < 0.05) between treatments or over time between intervals. Overall *p*-value for the interaction: *p* = 0.017.

There was a significant interaction between the foam and social treatments (*p* > 0.001) demonstrating that pigs placed in the foam box alone showed higher activity (i.e., number of floor squares crossed) than paired pigs, while there was no significant effect of social treatment in the controls without foam in the box (Figure 2). Moreover, pigs in the pair treatments (familiar and unfamiliar) were less active in the foam compared to the control treatments. There was a significant interaction between foam treatment and interval (*p* = 0.017), where pigs in the foam treatment showed a reduction in activity after the start of foam production (Figure 3, interval 5) and at the end of the test period (Figure 3, intervals 11 and 12). Pigs in the foam treatment were more active than pigs in the control treatment in interval 9, i.e., when the foam covered the foam-exposed pigs (Figure 3).

For escape attempts, there were no significant effects or interactions between effects in the model analysed. However, before foam production started, escape attempts were only performed by pigs placed in the box alone. Moreover, escape attempts were observed at least once during the total treatment duration (intervals 4–12) by 75% of the pigs in the Foam Alone treatment, while the corresponding figure was 42% in the Foam Unfamiliar pair treatment and 33% in the Foam Familiar pair treatment. Chi square tests of pairwise differences in percentage of pigs performing escape attempts between Foam Alone and the two pair foam treatments showed significant differences, with *p* < 0.001 for both, but no significant difference between the familiar and unfamiliar social treatments. The mean number of escape attempts during the entire time pigs were in the box (intervals 1–12) was 3.5 (range 0–13), 1.6 (range 0–9), and 0.9 (range 0–5) for Foam Alone, Foam Unfamiliar pair, and Foam Familiar pair treatment, respectively.

**Figure 4:**
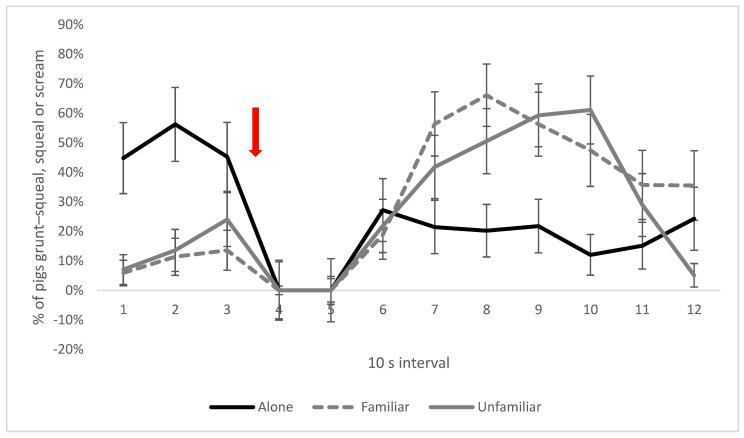
Percentage (%) of pigs vocalising with high-frequency grunt−squeals, squeals, or screams at least once per interval in the social treatments. Red arrow indicates time of foam start. Values given are LSM ± SE. Lack of overlap of SE error bars indicates significant pairwise difference (*p* < 0.05) between treatments or over time between intervals. Overall *p*−value for the interaction: *p* < 0.001.

A higher proportion of pigs placed in the foam box alone performed high-frequency vocalisations (grunt–squeals, squeals, and screams; Figure 4, intervals 1–3) directly after the pigs were placed in the box (before the onset of foam production) compared with pairs of pigs (interaction between social treatment and interval, *p* < 0.001). There was a clear decrease in the proportion of pigs performing high-frequency vocalisations during intervals 4 and 5, following the onset of foam production. Moreover, a lower proportion of single pigs emitted high-frequency vocalisations compared to the paired treatments during foam exposure (Figure 4, intervals 7–10). There were no significant treatment effects or interactions between treatments for low-frequency vocalisations (grunts).

**Figure 5:**
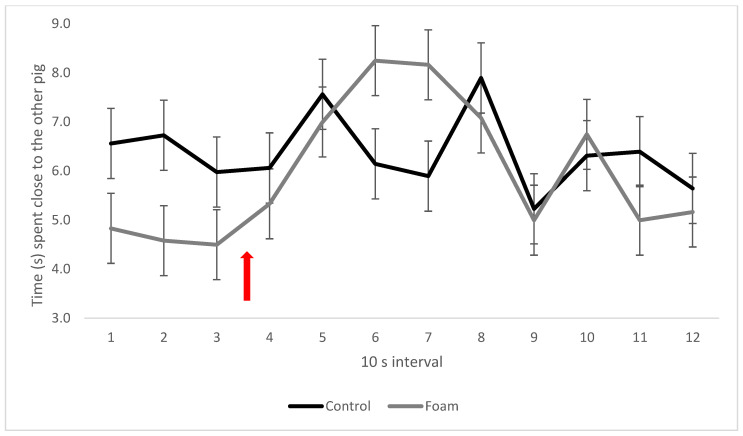
Pig closeness in the control and foam treatments, expressed as mean time (s) spent sharing a floor square with at least one hoof each per 10 s interval. Red arrow indicates time of foam start. Values given are LSM ± SE. Lack of overlap of SE error bars indicates significant pairwise difference (*p* < 0.05) between treatments or over time between intervals. Overall *p*-value for the interaction: *p* = 0.006.

**Figure 6:**
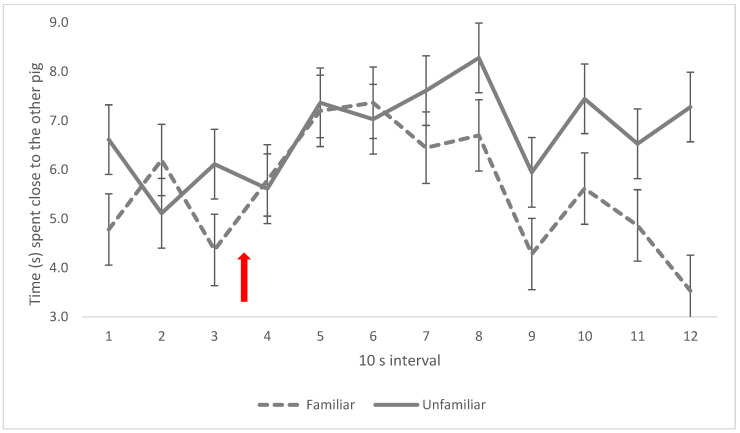
Pig closeness in the social treatments, expressed as mean time (s) spent sharing a floor square with at least one hoof each per 10 s interval. Red arrow indicates time of foam start. Values given are LSM ± SE. Lack of overlap of SE error bars indicates significant pairwise difference (*p* < 0.05) between treatments or over time between intervals. Overall *p*-value for the interaction: *p* = 0.019.

Pigs in the foam treatment increased their time close to each other as the foam level rose in intervals 6–7 (Figure 5) compared with controls (interaction between foam treatment and interval, *p* = 0.006). There was no difference in time spent close to another pig between familiar and unfamiliar pairs in the first 7 intervals in the box, but thereafter (intervals 8–12), familiar pigs reduced the time spent close to the other pig (Figure 6), while unfamiliar pigs did not (interaction between social treatment and interval, *p* = 0.019).

**Figure 7:**
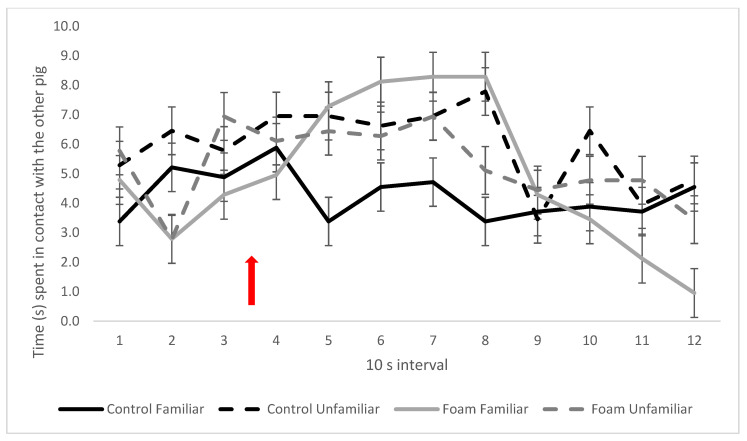
Pig contact, expressed as mean time (s) spent in physical contact with each other per interval in the four pair treatments. Red arrow indicates time of foam start. Values given are LSM ± SE. Lack of overlap of SE error bars indicates significant pairwise difference (*p* < 0.05) between treatments or over time between intervals. Overall *p*-value for the three-way interaction: *p* < 0.001.

Familiar pairs of pigs exposed to foam displayed more physical contact with each other during foam exposure in intervals 5–8 (Figure 7) compared with corresponding familiar control pairs. However, unfamiliar pairs of pigs behaved similarly regardless of treatment, with the only difference between treatments during foam exposure being more time spent in physical contact for the Control Unfamiliar pair in interval 8 (3-way interaction between foam treatment, social treatment, and interval, *p* < 0.001; Figure 7).

Agonistic behaviours were rare. During the observation period (intervals 1–12), only one test was scored “yes” on occurrence of agonistic behaviour (Control Unfamiliar pair), and three were scored “ambiguous” (one Control Familiar pair and two Control Unfamiliar pair). However, outside the observation period (i.e., intervals 1–12), two tests in the Control Unfamiliar pair treatment reached the humane endpoint, as the pigs engaged in prolonged fighting inside the box.

## 4. Discussion

In line with previous results [6], pigs in the present study placed in the foam box alone displayed behavioural responses related to aversiveness, as indicated by higher activity levels and more escape attempts. In contrast, the presence of a companion pig appeared to attenuate the response of pigs to the novel situation of foam exposure. There were interesting familiarity-related differences in the social behaviour displayed by pairs of pigs, suggesting, at least partly, different motivations for their behaviour.

Before the onset of foam production, escape attempts were only observed in single pigs, suggesting that being confined alone in the box was stressful for the pigs. The proportion of single pigs performing at least one escape attempt when exposed to foam (75%) was comparable to the 80% observed in the previous study [6].

When the pigs were exposed to the novel situation of foam inside the box, companionship attenuated the response, as indicated by the lower number of escape attempts and overall lower activity levels of pigs in pair foam treatments. During foam exposure, pairs of pigs spent more time close to each other as foam levels rose. This apparent preference for social proximity in a potentially stressful novel situation can be interpreted as a sign of social support [24,25,26]. However, only familiar pig pairs showed a marked difference in social behaviour based on treatment (Foam or Control). Pigs with a familiar pig present, spent more time in physical contact with the other pig during foam exposure, whereas unfamiliar pig pairs behaved similarly in both control and foam treatments. Unfamiliar pig pairs spent more time close to each other as time progressed in both the control and foam treatment, and only unfamiliar pig pairs had to be separated due to fighting (outside of the 2 min observation period). These differences based on familiarity or not between pigs indicate different motivations for the behaviour of familiar and unfamiliar pig pairs.

The motivation for the behaviour of unfamiliar pig pairs in this study could stem from a variety of causes, such as social interest, distraction, conflict, or support. Mixing of unfamiliar pigs is a known source of stress and fighting [23], and although previous studies have shown that piglets (7–35 days old) can derive social support from an unfamiliar piglet [12,15], it remains unclear whether this effect remains in older pigs. The occurrence of agonistic behaviour and fighting in the present study was, although sparse, only observed for control treatments, suggesting an underlying motivation of social distraction or conflict contributing to the social behaviour displayed by the unfamiliar pairs in foam.

It is obvious from the results that the unfamiliar pairs showed an interest in each other, as they spent a significant amount of time in social proximity and contact, regardless of the treatment. The emotional valence behind their social behaviour is ambiguous, though, since although some unfamiliar pig pairs fought, most interactions were clearly not agonistic. Furthermore, there was no significant difference between the familiar and unfamiliar pairs for escape attempts or activity, which would have been expected if the difference in the pigs’ distress levels was high. What stands out between the familiar and unfamiliar pig pairs regarding social support is that familiar pairs so clearly stayed in more physical contact during the acute phase of foam exposure, while unfamiliar pairs did not. Physical contact has been suggested as a major driver for the benefits of social support, as the attenuating effects of companionship are greatly improved by the possibility of touch [25,26]. It is also worth noting that because the pigs were confined in a small space inside the box, the measure of closeness might have been a less useful indicator of social support than physical contact in the present study, as the pigs always remained relatively close while inside the box. The fact that unfamiliar pig pairs remained close together also after foam exposure can have multiple explanations, as stated above (social interest, distraction, conflict, or support), and the only thing known for sure is that their motivation was something other than merely to seek social support from the foam exposure, as their behaviour otherwise would have been identical to that of familiar pig pairs.

Based on the results from the present study, it is possible that an unfamiliar pig might have contributed some level of social support during exposure to high-expansion air-filled foam, as the unfamiliar pairs showed the same attenuated behavioural response as familiar pairs regarding escape attempts and activity. However, the motivation behind the behaviours of unfamiliar pigs could also stem from social interest, distraction, or conflict, and we should be careful in attributing benefits from social support from a social setting where some pigs engage in fighting.

Companionship with another pig also influenced pig vocalisations. The decrease in high-frequency vocalisations, such as screams, detected after foam production began is in line with previous findings [6]. When exposed to the novel environment, the period before the onset of the foam production, a higher proportion of pigs in the alone treatment performed high-frequency vocalisations, indicating that social support altered the experience of the novel environment. However, during the later parts of the time the pigs were kept in the foam box, a higher proportion of pigs with a companion performed high-frequency vocalisations, which could indicate that the pigs trigged each other or sought social support. The greater expression of high-frequency vocalisations in pairs of pigs seems to contradict the favourable effect of social support, as these types of vocalisations are associated with a negative emotional state [27]. This may be a side effect of emotional contagion when the individual offering social support also is distressed. There was also a higher likelihood of vocalisations in pair treatments to begin with, as these by design had a double number of pigs.

The emotional state of an animal affects others in the group, and this emotional contagion seems especially strong in conveying negative emotions [28]. From an evolutionary perspective, it probably served social animals well to be easily influenced by the emotional status of others, as this was a possible indicator of life-threatening danger. The ability of a companion to successfully deliver social support to a distressed conspecific is thus dependent on its own emotional state. A distressed individual is in general a poor social supporter, and it is much better from an animal welfare point of view if the companion is a calm, low-stress individual [16,29,30]. Previous research indicates that emotional contagion is enhanced between familiar individuals [31], which is important to consider when comparing the effect of social support from a familiar and unfamiliar pig companion. However, the present study showed no difference between familiar and unfamiliar pairs in overall activity level or escape attempts, suggesting that a familiar companion did not clearly amplify the pig’s distress levels through a greater emotional contagion. Considering the effects of emotional state on emotional contagion and social support, lowering the overall stress levels of pigs before and during stunning at slaughter could enhance animal welfare at both the individual and group level.

The time of mixing unfamiliar pigs before or during transport to slaughter and at the abattoir likely influences their ability to derive social support from each other at stunning. SThe unfamiliar pigs in the present study did not start to fight until a few minutes inside the foam box, and the results might have been different if the unfamiliar pig pairs had been introduced to each other hours or days before being placed in the foam box. However, mixing is known to cause stress and injury in pigs and should be avoided, especially with older, mature slaughter pigs, who tend to show more aggression than younger pigs [32,33].

As the study only included the behavioural response of mixed-sex paired weaner pigs, we cannot generalise the results to older, mature slaughter pigs during the novel situation of foam exposure. This study is limited to the interpretation of the behavioural responses of the pigs due to the practical and ethical challenges in measuring physiological parameters in pigs kept with conspecifics. Using non-invasive equipment to measure physiological parameters in groups of pigs is difficult, as the curious pigs manipulate the equipment. Moreover, collecting blood samples would require restraining, which would likely be more stress-inducing than the novel situation under study, thus not contributing to the comparison of stress levels between the studied treatments. The use of weaners was supported by the ease of comparing the results with previous studies on high-expansion foam exposure with weaner pigs [6]. Studying pairs of weaner pigs was also due to the practical space limitations of the available foam box equipment, as well as a precautionary measure for studying the effect of unfamiliar pairs, as fighting in older pigs would have increased the risk of injury to the pigs. The decision to use mixed-sex pairs was based on the lower number of animals needed, compared to a set-up studying all sex-pair combinations, as well as the lack of necessity in doing so with weaners, with small differences in behaviour between sexes.

## 5. Conclusions

Weaner pigs showed behavioural responses in the form of increased activity and escape attempts when exposed to high-expansion air-filled foam inside a foam box. The companionship of another pig decreased the pigs’ activity and number of escape attempts, and the pairs also stayed closer together during foam exposure. Familiar pig pairs spent more time in physical contact during foam exposure, while unfamiliar pairs spent more time close to each other as time progressed in the box, regardless of treatment. Unfamiliar pairs occasionally also started to fight while inside the box. The motivation behind the behaviour of unfamiliar pairs is discussed with regards to social interest, distraction, conflict, and support, and although we are cautious to imply social support benefits from a social setting where some pigs may fight, the possibility cannot be excluded based on the present results. The findings obtained in this study highlight the effects of familiarity among weaner pigs in social support and suggest pigs seek social support from a familiar pig through physical contact during a novel situation. The results also demonstrate the importance of considering the effect of social support when evaluating and aiming to improve the welfare of social animals.

## Figures and Tables

**Table 1:** Pig body weights and weight differences between pairs of pigs within the treatments (Control Alone, Control Familiar pair, Control Unfamiliar pair, Foam Alone, Foam Familiar pair, and Foam Unfamiliar pair). Mean value and standard deviation (Std).

Treatment	Weight (kg)		Weight Difference (kg)	
	Mean	Std	Mean	Std
Control Alone (*n*= 12)	31.5	2.8	-	-
Control Familiar pair (*n* = 12)	29.0	4.1	3.5	2.5
Control Unfamiliar pair (*n* = 12)	30.2	3.1	2.9	1.6
Foam Alone (*n* = 12)	32.3	6.8	-	-
Foam Familiar pair (*n* = 12)	30.1	5.2	7.1	4.0
Foam Unfamiliar pair (*n* = 12)	32.0	3.4	4.0	3.3
Total (*n* = 72 Weight, *n* = 48 Weight difference)	30.8	4.5	4.4	3.5

**Table 2:** Ethogram used for observations of pig behaviour inside the foam box. Activity and escape attempts were recorded on an individual pig basis. Vocalisations and social behaviours were recorded on a pig pair basis.

Behaviour	Definition	Registration
*Activity*		
Locomotion	Number of floor squares crossed with both front hooves	Continuous recording of frequency within 10 s intervals
*Escape attempts*		
Escape door	Jumping at, pushing against, or kicking at the door	Continuous recording of frequency within 10 s intervals
Escape roof	Jumping at or pushing against the roof	
Escape wall	Jumping at, pushing against, or kicking at the walls	
*Vocalisations*		
Low frequency	0 = no grunts 1 = ≤3 grunts 2 = >3 grunts	Score per 10 s interval
High frequency	0 = no grunt–squeals, squeals, or screams 1 = ≤3 grunt–squeals or squeals 2 = screams and/or >3 grunt–squeals or squeals	
*Social behaviour*		
Closeness	Both pigs have at least one hoof each in the same floor square	Continuous recording of duration within 10 s intervals
Contact	Physical contact with conspecific	
Agonistic	Pushing with force against, biting, or engaging in fighting behaviour with conspecific	Scored as “yes”, “no”, or “ambiguous” per test

Categories of behaviours shown in italic.

## Data Availability

The data presented in this study are available in the supplementary materials.

## Supplementary Materials

Supporting information in the form of a raw data file in Excel can be downloaded at: https://www.mdpi.com/article/10.3390/ani13030481/s1.
